# Parkin Protects against Oxygen-Glucose Deprivation/Reperfusion Insult by Promoting Drp1 Degradation

**DOI:** 10.1155/2016/8474303

**Published:** 2016-08-14

**Authors:** Jiayu Tang, Zhiping Hu, Jieqiong Tan, Sonlin Yang, Liuwang Zeng

**Affiliations:** ^1^Department of Neurology, Second Xiangya Hospital, Central South University, Changsha, Hunan 410011, China; ^2^Department of Neurology, The Second People's Hospital of Hunan Province, Changsha, Hunan 410007, China; ^3^National Key Laboratory of Medical Genetics, Central South University, Changsha, Hunan 410078, China

## Abstract

Ischemic stroke results in severe brain damage and remains one of the leading causes of death and disability worldwide. Effective neuroprotective therapies are needed to reduce brain damage resulting from ischemic stroke. Mitochondria are crucial for cellular energy production and homeostasis. Modulation of mitochondrial function mediates neuroprotection against ischemic brain damage. Dynamin-related protein 1 (Drp1) and parkin play a key role in regulating mitochondrial dynamics. They are potential therapeutic targets for neuroprotection in ischemic stroke. Protective effects of parkin-Drp1 pathway on mitochondria were assessed in a cellular ischemia-reperfusion injury model. Mouse neuroblastoma Neuro2a (N2a) cells were subjected to oxygen-glucose deprivation/reperfusion (OGDR) insult. OGDR induces mitochondrial fragmentation. The expression of Drp1 protein is increased after OGDR insult, while the parkin protein level is decreased. The altered protein level of Drp1 after OGDR injury is mediated by parkin through ubiquitin proteasome system (UPS). Drp1 depletion protects against OGDR induced mitochondrial damage and apoptosis. Meanwhile, parkin overexpression protects against OGDR induced apoptosis and mitochondrial dysfunction, which is attenuated by increased expression of Drp1. Our data demonstrate that parkin protects against OGDR insult through promoting degradation of Drp1. This neuroprotective potential of parkin-Drp1 pathway against OGDR insult will pave the way for developing novel neuroprotective agents for cerebral ischemia-reperfusion related disorders.

## 1. Introduction

Mitochondria, the power house of the cell, participate in many essential cellular functions, including energy production, ion homeostasis, inflammation, apoptotic cell death, and calcium signaling. Change in mitochondrial mass and function has been linked with multiple diseases, including cerebral ischemia. Mitochondrial dysfunction is the most fundamental mechanism of cell damage in cerebral ischemia-reperfusion injury, which involves multiple independently fatal terminal pathways in the mitochondria. Modulation of mitochondrial function mediates neuroprotection against ischemic brain damage. Mitochondria are promising targets for stroke therapy [1, 2].

Mitochondrial homeostasis depends on their biogenesis and degradation. Parkin and dynamin-related protein 1 (Drp1) play a pivotal role in mitochondrial fission and clearance [3]. Parkin, the ubiquitin E3 ligase, has been shown to control the biogenesis and degradation of mitochondria. Parkin has also been suggested to ubiquitinate mitochondrial proteins, such as Drp1, to promote autophagy of damaged mitochondria [4]. Drp1 is required for mitochondrial division in mammalian cells. Changes in Drp1 expression directly influence cellular metabolism and ultimately cell fate. Drp1 is required for functionally active mitochondria, and supplementing with ATP can restore the defects induced by Drp1 suppression [5]. Drp1 is activated after cardiac arrest and the inhibition of Drp1 is protective against cerebral ischemic injury [6]. Parkin and Drp1 are novel therapeutic targets for cytoprotection.

Therefore, on the basis of previous findings, we presumed that parkin and Drp1 would exert neuroprotective effect on cerebral ischemia/reperfusion that occurred in stroke. To address this, we employed oxygen-glucose deprivation and reperfusion (OGDR) model, which had been widely used in cultured neurons and brain slices to simulate brain ischemia. We found that Drp1 depletion protects against OGDR induced mitochondrial damage and apoptosis. Meanwhile, overexpression of parkin protects against OGDR induced apoptosis and mitochondrial dysfunction, which is blocked by upregulation of Drp1. Thus, parkin-Drp1 pathway represents a novel therapeutic target for treatment of a myriad of disorders related to cerebral ischemia-reperfusion injury.

## 2. Materials and Methods

### 2.1. Cells Culture and Transfection

Mouse N2a neuroblastoma cells were purchased from American Type Culture Collection (ATCC). N2a neuroblastoma cells were used and maintained in Dulbecco's modified Eagle's medium (DMEM), supplemented with 10% FBS (Gibco BRL), 100 U/mL penicillin, and 100 *μ*g/mL streptomycin, at 37°C in a moist atmosphere containing 5% CO_2_. For overexpression of parkin and Drp1, cells were transfected with the indicated plasmids (pcDNA3.1-parkin-myc and pcDNA3.1-Drp1-HA) using Lipofectamine 2000 (Invitrogen) according to manufacturer's instructions.

### 2.2. Oxygen-Glucose Deprivation and Reperfusion (OGDR)

To mimic ischemic-like conditions in vitro, cell cultures were exposed to oxygen-glucose deprivation (OGD) for 4 hours and then returned to 95% air, 5% CO_2_, and glucose-containing medium for different recovery time as before. First, mouse N2a neuroblastoma cells were transferred into a temperature controlled (37°C) anaerobic chamber (Forma Scientific) containing a gas mixture composed of 5% CO_2_ and 95% N_2_. The culture medium was replaced with deoxygenated glucose-free Hanks' Balanced Salt Solution (Invitrogen) and cells were maintained in the hypoxic chamber for 4 hours. After OGD, N2a cells were maintained in DMEM supplemented with 10% FBS under normoxic culture conditions for 0, 4, and 12 hours.

### 2.3. Immunofluorescence Staining

Mouse N2a neuroblastoma cells were subjected to OGD for 4 hours followed by reperfusion for 0, 4, and 12 hours. The cells were then fixed with 4% paraformaldehyde for 30 min and washed three times with PBS, pH 7.4. The cells were incubated with a primary rabbit anti-Tom20 antibody (1 : 200; SC-11415, Santa Cruz Biotechnology) overnight at 4°C. On the following day, the cells were incubated with fluorescein-conjugated anti-rabbit IgG (1 : 400; FI-1000, Vector Laboratories) for 1 h. N2a cells were counterstained with 1 *μ*g/mL 4′,6-diamidino-2-phenylindole (DAPI) (Vector Laboratories, Burlingame, CA, USA) to visualize nuclear morphology. Slides were washed, wet-mounted, and observed using a confocal microscope (Zeiss LSM Meta710, 63x/1.4 objective). Mitochondria fragmentation was assessed visually and quantified as percentage of control. Cells that displayed a network of filamentous mitochondria were classified as normal. Cells with fragmented or partially reelongated mitochondria were classified as fragmented [7].

### 2.4. Western Blot Analysis

Total protein was isolated from the N2a cells using 2x SDS sample buffer (63 mM Tris-HCl, 10% glycerol, and 2% SDS). Samples (20–40 *μ*g of protein) were electrophoresed onto a 10–15% SDS/polyacrylamide gel (SDS/PAGE) and transferred to PVDF membranes. The membranes were blocked in TBS-Tween buffer containing 20 mM Tris-HCl, 5% nonfat milk, 150 mM NaCl, and 0.05% Tween-20 (pH 7.5) for 1 hour at room temperature. Thereafter, the blot was incubated with primary parkin mouse monoclonal antibody (1 : 1000; P-6248, Sigma-Aldrich), Drp1 rabbit monoclonal antibody (1 : 1000, #8570, cell signaling), Myc-tag mouse monoclonal antibody (1 : 1000; ab18185, Abcam), and actin mouse monoclonal antibody (1 : 10,000; SC-47778, Santa Cruz) for 1-2 hours at room temperature. The membrane was washed with TBST 3 times at 10-minute intervals, incubated with the secondary antibody (1 : 5000; anti-rabbit or anti-mouse IgG conjugated with horseradish peroxidase; Jackson ImmunoResearch Laboratories) at room temperature for 1 hour, and then washed 3 times each at 10-minute interval with TBST and 2 times each for 10 minutes with TBS. Band was visualized via an enhanced chemiluminescence kit (ECL) according to the manufacturer's suggested protocol (GE Health). Membranes were then exposed to X-ray film.

### 2.5. Quantitative Real-Time PCR

Total RNA was isolated from N2a cells by using TriZol (Invitrogen). Reverse transcription was performed by using the Reverse Transcription Kit (Promega). Equal amounts of total RNA (500 ng) were reverse-transcribed. Primers for Drp1 and actin were used as follows: Drp1 forward primer, 5′-ACAGGAGAAGAAAATGGAGTTTGAAGCAG-3′, Drp1 reverse primer, 5′-AACAAATCCTAGCACCACGCAT-3′, actin forward primer 5′-AGGCACCAGGGCGTGAT-3′, and actin reverse primer, 5′-GCCCACATAGGAATCCTTCTGAC-3′. Quantitative real-time PCRs were then conducted at 95°C for 10 s, followed by 40 cycles of 95°C for 5 seconds and 60°C for 30 s. The relative RNA levels were normalized to endogenous actin expression for each sample. The PCR experiments were repeated 3 times, each using separate sets of cultures.

### 2.6. siRNA-Mediated Parkin/Drp1 Knockdown

Drp1 siRNA and control siRNA were used for knocking down protein expression (sequence: siDrp1: 5′-AAGCAGAAGAAUGGGGUAAAUdTdT-3′ and siparkin: 5′-UUCCAAACCGGAUGAGUGGdTdT-3′). The N2a neuroblastoma cells were transiently transfected with siRNA against Drp1 or parkin synthesized by GenePharma using Lipofectamine 2000 reagent (Invitrogen) before OGD. The scrambled sequence was used as a negative control. Lipofectamine 2000 (Invitrogen, Germany) and siRNA were dissolved separately in Optimem I (Invitrogen). After standing at room temperature for 10 min, each RNA solution was added with the Lipofectamine 2000 and mixed for 20 min gently allowing formation of siRNA liposomes at room temperature. Then the mixture was added to the DMEM (antibiotic-free) to a final concentration of 60 nmol and 2 *μ*L/mL Lipofectamine. Protein expression was determined after 48 hours of transfection. The OGD was performed 48 h after transfection.

### 2.7. Measurement of Apoptosis

Mouse N2a neuroblastoma cells were subjected to OGD for 4 hours followed by reperfusion for 12 hours. Apoptosis was detected by Annexin V FITC Apoptosis Detection Kit (Sigma). Briefly, N2a cells were collected and washed twice with PBS. 500 *μ*L binding buffer suspension was then added to the treated cells. After that, 5 *μ*L Annexin V-FITC and 10 *μ*L propidium iodide were added to each group and cultures were incubated at 37°C for 5~15 min in dark. Flow cytometer (BD Biosciences) was used to detect the percent cells with apoptosis and flowJo software was used for flow cytometry analysis.

### 2.8. Cytochrome c Oxidase (COX) Activity Assays

Mitochondria of N2a cells were isolated using the animal cells/tissues mitochondria extraction kit (Genmed Scientifics, Cat. GSM10006). Cytochrome c oxidase activity was detected using cytochrome c oxidase assay kit (Sciencell, Cat. 8278). All procedures followed the manufacturer's instructions. Briefly, mitochondria were suspended in 50 *μ*L of isolation buffer containing 250 mM sucrose, 20 mM HEPES, pH 7.2, and 1 mM EDTA. Suspensions were added to a cuvette containing 0.95 mL of 1x assay buffer (10 mM Tris-HCl, pH 7.0, and 120 mM KCl), and the reaction volume was brought to 1.05 mL with 1x enzyme dilution buffer (10 mM Tris-HCl, pH 7.0). The reaction was then started by the addition of 50 *μ*L of cytochrome c substrate solution; the change in absorbance of cytochrome c at 550 nm was measured using a DU640 spectrophotometer. The reading was recorded every 5 sec during the first 3 minutes. Background levels were measured without cell suspensions. All measurements were repeated at least three times. All normalized control of cytochrome c oxidase activity values will be 1 (or 100%).

### 2.9. Determination of Mitochondrial ATP Synthase Activity

Mitochondrial ATP synthase activity was detected as follows. Mitochondria of N2a cells were isolated using the animal cells/tissues mitochondria extraction kit (Genmed Scientifics, Cat. GSM10006). Ten micrograms of mitochondria was used to detect ATP synthase activity by using the mitochondrial complex V activity quantitative analysis kit (Genmed Scientifics, Cat. GMS50083). All procedures followed the manufacturer's instructions. Briefly, the ADP generated by complex V reacts with phosphoenolpyruvic acid (1 mM) to pyruvate in the presence of pyruvate kinase (1.5 U/mL). Pyruvate oxidizes with NADH (250 *μ*M) to lactate and NAD+ in the presence of lactate dehydrogenase (2.5 U/mL). This reaction was monitored spectrophotometrically at 340 nm for 8 min and used to determine complex V activity. All measurements were repeated at least three times. All normalized control of complex V activities values will be 1 (or 100%).

### 2.10. Statistical Analysis

Quantitative data were expressed as mean ± SEM based on at least 3 separate experiments of triplicate samples. Differences among groups were statistically analyzed by one-way analysis of variance followed by Bonferroni's post hoc test. Comparison between two experimental groups was based on a two-tailed *t*-test. Differences between the mean values were considered significant if *p* < 0.05.

## 3. Results

### 3.1. OGDR Induces Mitochondrial Fragmentation in N2a Cells

**Figure 1:**
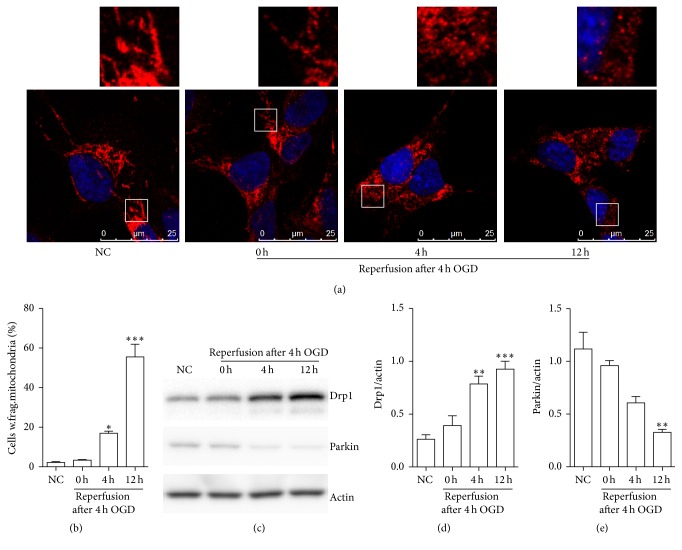
OGDR affects mitochondrial morphology and the protein levels of Drp1 and parkin. (a) Mitochondrial morphology was analyzed with Tom20 staining by confocal microscopy in N2a cells. The confocal images and the enlarged section of the confocal images are displayed. (b) The average percentage of N2a cells with fragmented mitochondria was measured. (c) Western blot analysis and (d, e) quantitative analysis of Drp1 and parkin expressions in N2a cells upon OGDR insult. Actin was used as a loading control. Data are presented as the mean ± SEM. Asterisks indicate statistically significant difference compared with the control; ^*∗*^ *p* < 0.05, ^*∗∗*^ *p* < 0.01, and ^*∗∗∗*^ *p* < 0.001.

To explore whether mitochondrial fragmentation occurs in N2a cells upon OGDR insult, we used immunofluorescent staining to evaluate its temporal profiles (Figure 1). The increase of mitochondrial fragmentation in a time-dependent manner was found during the different time points of OGDR. As demonstrated in Figure 1(a), most of the cells displayed tubular and long mitochondria in normal conditions, indicating a balance between mitochondrial fusion and fission. After 4 h of OGD treatment, most of the cells still showed tubular and long mitochondria. However, after 4 and 12 h reperfusion following 4 h of OGD, the morphology of mitochondria changed to debris-like structures scattered in the cytoplasm. The increase of N2a cells with fragmented mitochondria began as early as 4 h reperfusion following 4 h OGD exposure and was further enhanced after 12 h reperfusion (Figure 1(b)).

### 3.2. Expression Pattern of Drp1 and Parkin Protein in N2a Cells upon OGDR Insult

To elucidate the functional significance of Drp1 and parkin in mouse N2a cells upon OGDR challenge, we first determined the expression profile of Drp1 and parkin protein at different reperfusion time points following OGD insults. Western blot analysis showed similar expression of Drp1 between normal mouse N2a cells and N2a cells after 4 h of OGD. However, expression of Drp1 was strongly increased in mouse N2a cells after 4 and 12 h reperfusion following 4 h of OGD (Figures 1(c) and 1(d)). Meanwhile, western blot analysis demonstrated that the protein level of parkin was decreased in mouse N2a cells after OGDR insult, which was most significant after 12 h reperfusion following 4 h of OGD (Figures 1(c) and 1(e)).

### 3.3. OGDR Regulates the Expression of Drp1 Protein via Inhibition of Ubiquitin Proteasome System

**Figure 2:**
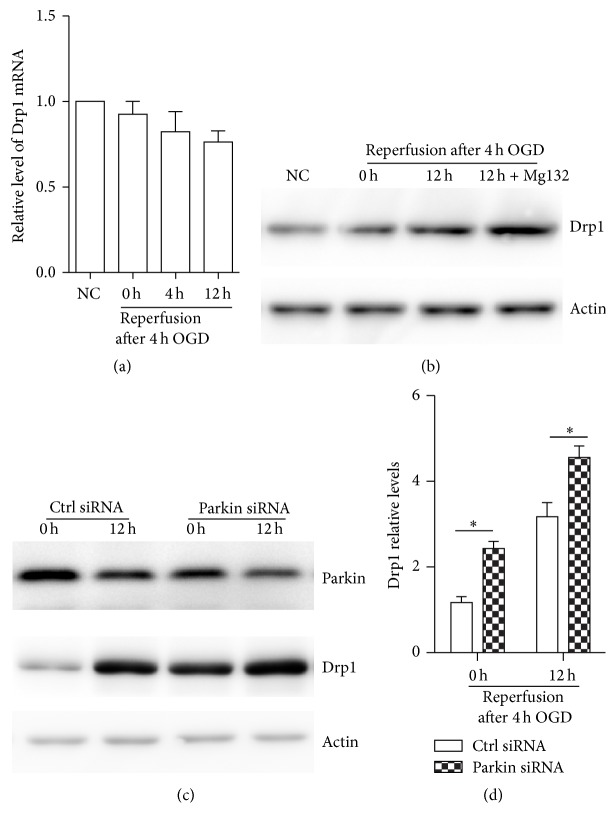
OGDR affects Drp1 expression through ubiquitin proteasome system. (a) mRNA level of Drp1 in mouse N2a cells upon OGDR insult was assessed by quantitative real-time PCR. Actin mRNA was used as an internal control. (b) Mouse N2a cells were incubated in the absence or presence of MG132, a reversible proteasome inhibitor. Cell lysates were analyzed by western blot with antibodies against mitochondrial fission-specific Drp1. Actin was used as a loading control, and all blots are representative of three independent experiments. (c) N2a cells were transfected with parkin siRNA or control siRNA (Ctrl siRNA), and western blot was performed to examine the expression of parkin and Drp1 protein. (d) Drp1 protein level was quantified according to the results of three independent blots. Data represent the mean ± SEM. Asterisks indicate a statistically significant difference compared with the control. ^*∗*^ *p* < 0.05.

We next examined whether OGDR regulated Drp1 at the mRNA level in mouse N2a cells. Quantitative real-time PCR demonstrated that Drp1 transcription was unchanged in normal mouse N2a cells and N2a cells subjected to 0, 4, and 12 h reperfusion following 4 h of OGD, indicating that OGDR regulating the expression of Drp1 is not transcriptional (Figure 2(a)).

We next used the proteasome inhibitors MG132 (20 *μ*M for 4 h) to determine whether the OGDR-mediated increase in Drp1 protein expression was due to inhibition of ubiquitin proteasome system (UPS). We found that the expression of Drp1 protein was significantly increased after treatment with the MG132 (Figure 2(b)). Taken together, these data demonstrate that OGDR induces Drp1 expression through the proteasome-dependent proteolytic machinery.

### 3.4. Parkin Promotes Drp1 Degradation after OGDR Insult

Parkin, an E3 ubiquitin ligase, can regulate the ubiquitination and proteasome-dependent degradation of its substrates. The Drp1 protein level has been reported to be regulated by the E3 ubiquitin ligase, parkin [8, 9]. We further investigated the regulatory effect of parkin on Drp1 expression after OGDR insult. siRNA-mediated knockdown of parkin expression was performed in mouse N2a cells. We found that Drp1 protein expression was significantly increased after knockdown of parkin (Figures 2(c) and 2(d)), suggesting that parkin induces Drp1 degradation after OGDR injury.

### 3.5. Knockdown of Drp1 Protects against OGDR Induced Mitochondrial Damage and Apoptosis

**Figure 3:**
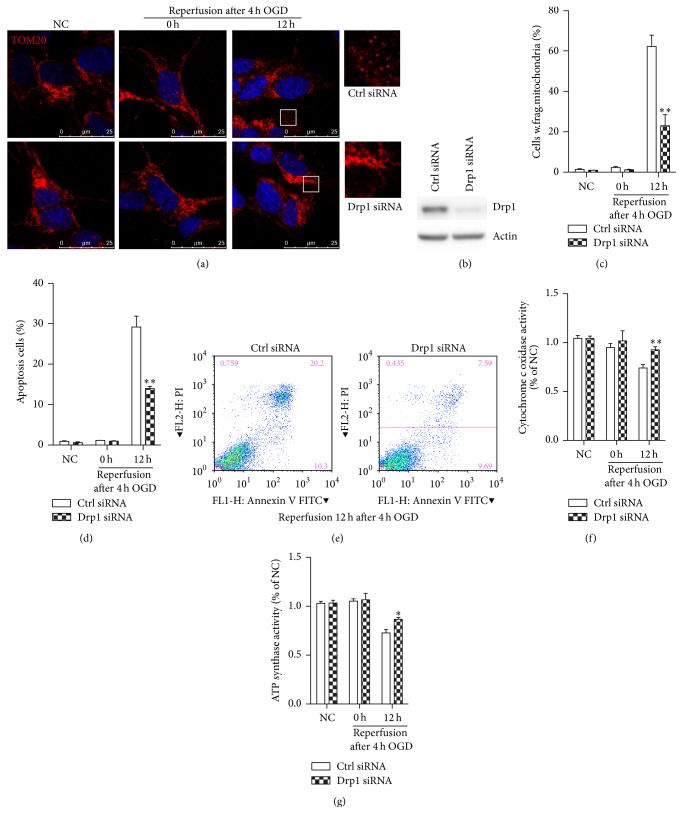
The effect of Drp1 knockdown on mitochondria morphology, function, and apoptosis in N2a cells exposed to OGDR insult. After being transfected with siRNA against Drp1, cells were treated with OGD 4 h plus 12 h reperfusion. The experiment was repeated independently for at least three times. (a) Digital photomicrograph under fluorescent illumination showing the morphology of mitochondria was detected using Tom20 staining. The confocal images and the enlarged section of the confocal images are displayed. Most of N2a cells displayed typical tubular and long mitochondria in normal condition or after 4 h OGD. Fragmented mitochondria were evident in N2a cells exposed to 4 h OGD plus 12 h reperfusion. Transfection with siRNA against Drp1 significantly attenuated OGDR induced fragmentation of mitochondria. (b) Representative immunoblots of Drp1 showed knockdown of Drp1 by the specific Drp1 siRNA. (c) Quantitation (mean ± SEM) of A from three independent experiments. (d, e) Knockdown of Drp1 in vitro reduced OGDR induced apoptosis. (f, g) Cytochrome c oxidase and mitochondrial ATP synthase activity in N2a cells with or without Drp1 depletion after OGDR insult. Drp1 protein knockdown significantly improved cytochrome c oxidase activity and mitochondrial ATP synthase activity after OGDR insult. Values are expressed as mean ± SEM. ^*∗*^ *p* < 0.05 and ^*∗∗*^ *p* < 0.01 compared to control.

To further analyze the role of Drp1 in ischemic injury, we examined the effect of Drp1 knockdown with a specific siRNA on OGDR induced mitochondrial damage and apoptosis in N2a cells. After being transfected with siRNA against Drp1, N2a cells were treated with OGD 4 h plus 12 h reperfusion. The RNA interference of Drp1 significantly reduced Drp1 protein levels in N2a cells (Figure 3(b)). Results showed that, in N2a cell without OGDR exposure, N2a cell displayed typical tubular and long mitochondria (Figure 3(a)). When N2a cell transfected with control siRNA is exposed to OGD 4 h plus 12 h reperfusion, lots of mitochondria changed to debris-like structures scattered in the cytoplasm. However, transfection with Drp1 siRNA significantly attenuated OGDR induced mitochondrial fragmentation (Figure 3(a)). Quantitative analysis demonstrated that there were a higher proportion of longer and tubular mitochondria in the cells treated with Drp1 siRNA compared with the vehicle-treated cells (Figure 3(c)). Additionally, the proportion of apoptotic cells was increased after OGD 4 h plus 12 h reperfusion. However, N2a cells transfected with Drp1 siRNA displayed a significant decrease in the number of apoptotic cells after OGDR exposure (Figures 3(d) and 3(e)).

Meanwhile, COX activity and mitochondrial ATP synthase activity were also measured in N2a cells to provide an assessment of mitochondrial function. The COX activity was decreased after OGDR injury. However, Drp1 depletion significantly attenuated OGDR induced decrease in activity of cytochrome c oxidase (Figure 3(f)). In addition, mitochondrial ATP synthase activity was measured to investigate the effect of Drp1 depletion on mitochondrial ATP generation in N2a cells. Mitochondrial ATP synthase activity (Figure 3(g)) was inhibited by OGDR treatment. However, ATP synthase activity was improved after being transfected with Drp1 siRNA. Together, these data strongly suggest that OGDR induced mitochondrial damage and apoptosis were markedly reversed by Drp1 knockdown.

### 3.6. Parkin Protects against OGDR Induced Apoptosis and Mitochondrial Dysfunction by Promoting Drp1 Degradation

**Figure 4:**
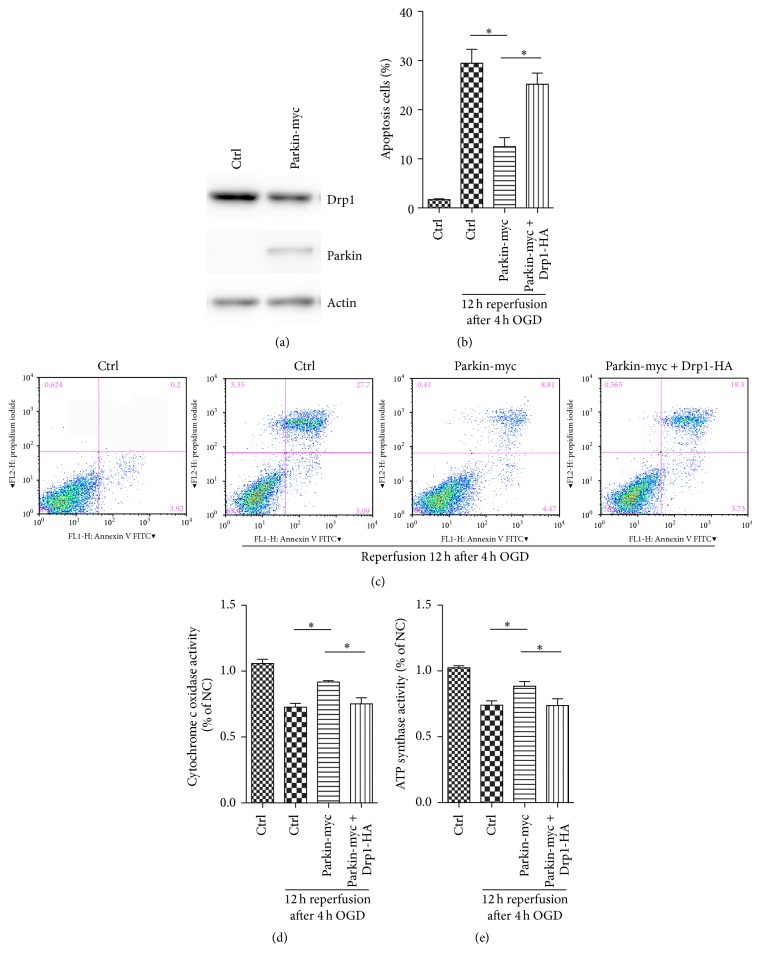
Inhibition of Drp1 by parkin overexpression confers protection against OGDR induced apoptosis and mitochondrial dysfunction. (a) Mouse N2a cells were untransfected or transfected with parkin-myc or vector, and western blot was performed to examine the expression of Drp1 and parkin proteins. Overexpression of parkin contributed to decreased expression of Drp1. (b, c) Mouse N2a cells untransfected or transfected with parkin-myc and Drp1-HA were subjected to 4 h OGD plus 12 h reperfusion. Enhanced expression of parkin was able to suppress OGDR induced apoptosis, which was abrogated by increased expression of Drp1. (d, e) Parkin overexpression improved cytochrome c oxidase and mitochondrial ATP synthase activities after OGDR injury, which was also attenuated by increased expression of Drp1. All data are representative of at least three independent experiments. Values are expressed as mean ± SEM. ^*∗*^ *p* < 0.05 compared to control.

We have demonstrated that parkin knockdown induced Drp1 expression after OGDR injury. To assess the role of parkin in the regulation of apoptosis and mitochondrial function after OGDR insult, we increased parkin expression in mouse N2a cells with specific expression plasmids for parkin with a Myc-tag. Parkin overexpression resulted in decreased expression of Drp1 protein (Figure 4(a)). Strikingly, enhanced expression of parkin was able to suppress OGDR induced apoptosis. However, parkin overexpression mediated protection against OGDR induced apoptosis was attenuated by increased expression of Drp1 (Figures 4(b) and 4(c)). Additionally, our results also demonstrated that the decreased activities of cytochrome c oxidase and mitochondrial ATP synthase after OGDR insult were improved after enhanced expression of parkin, which was abrogated by Drp1 overexpression (Figures 4(d) and 4(e), resp.). Therefore, our data suggest that the neuroprotective effect of parkin in OGDR insult is mediated by promoting Drp1 degradation.

## 4. Discussion

Ischemic stroke remains one of the leading causes of death and disability worldwide. Cerebral ischemia and subsequent reperfusion result in cellular organelles damage, which plays a pivotal role in the development of tissue injury after acute ischemic stroke. Ischemia-reperfusion insult generally leads to mitochondrial dysfunction and fragmentation [10, 11]. Consistent with previous finding, we also found mitochondrial fragmentation in N2a cells after 4 h and 12 h reperfusion following 4 h OGD exposure. Our data suggest that mitochondria are sensitive to OGDR insult. OGDR induced mitochondrial fragmentation would lead to mitochondrial dysfunction, such as failure in the energy production for cell homeostasis and cell survival in N2a cells. Mitochondrial dysfunction after OGDR injury is one of the major contributors to ischemia-reperfusion damage. Many agents confer neuroprotective effects by targeting mitochondria [12, 13]. Therefore, developing novel therapeutic strategies targeting mitochondrial pathway will open a new avenue for treating cerebral ischemia.

Moreover, OGDR insult not only induces mitochondrial fragmentation, but also regulates Drp1 expression. We found that the protein level of Drp1 was strongly upregulated after ODGR injury, while its transcription was unchanged, indicating that OGDR regulates the expression of Drp1 via a nontranscriptional mechanism. Mitochondrial morphology depends on the balance between two opposing processes, mitochondrial fission and fusion. Mitochondrial fission is mediated predominantly by a dynamin-related GTPase, Drp1 [14]. Thus, induced expression of Drp1 after OGDR injury could influence mitochondrial fission and functional activity in N2a cells. Drp1 upregulation would also influence cellular metabolism and ultimately cell fate after OGDR insult. In neurons, Drp1 controls the migration and neuronal differentiation of subventricular zone-derived neural progenitor cells [5]. Drp1-mediated mitochondrial fission plays a major role in neuronal cell death associated with acute ischemic brain damage [15]. Therefore, the changed expression of Drp1 may play a key role in the pathophysiology of OGDR insult.

In our study, we also demonstrated that OGDR insult inhibits parkin protein expression in mouse N2a cells, which was more remarkable after prolonged reperfusion period following 4 h of OGD. Parkin is a widely expressed 465 amino acid protein that possesses E3 ubiquitin-protein ligase activity [16]. Parkin plays a critical role in the pathogenesis of Parkinson's disease (PD) [17]. Loss-of-function mutations in the parkin gene have been revealed to be the most common cause of autosomal recessive, early-onset PD [18, 19]. For ischemic stroke, expression of parkin was also decreased in transient focal cerebral ischemia [20], which is in line with our results. Parkin exerts various roles in neurons and is widely thought to be protective. Decreased expression of parkin would contribute to mitochondria injury and tissue damage after OGDR insult. Mutations in parkin would result in ubiquitin proteasome system (UPS) dysfunction [21]. Parkin protein depletion after cerebral ischemia could increase the aggregation of ubiquitylated proteins and the sensitivity of neurons to endoplasmic reticulum (ER) dysfunction [20]. OGDR induced parkin inhibition would cause dysfunction of UPS and influence degradation of ubiquitylated proteins. Decreased parkin protein expression may be of great importance in the pathological processes culminating in neuronal cell injury after OGDR insult and ischemic stroke therapy.

Additionally, we found that expression of Drp1 protein was significantly enhanced after proteasome inhibitor MG132 treatment, indicating that OGDR-mediated increase in Drp1 protein expression is due to the inhibition of UPS. The UPS plays an important role in protein quality control, growth and inflammatory cell signaling, and neuron excitability. It has emerged as a drug target for diverse diseases characterized by altered proteostasis [22]. Thus, UPS might also be involved in OGDR insult induced cerebral damage. Meanwhile, we demonstrated that Drp1 protein expression was increased after parkin depletion after OGDR injury. Drp1 is a substrate of parkin. Parkin interacts with and subsequently ubiquitinates Drp1, for promoting its proteasome-dependent degradation [8]. Activation of the UPS for proteolysis of multiple outer membrane proteins is supposed to be a major function of parkin in dysfunctional mitochondria, which is a critical step in parkin-mediated mitophagy [23]. Therefore, parkin is involved in elimination of Drp1 after OGDR injury. Parkin uses the UPS to degrade and ship off Drp1 and consequently contribute to regulation of cellular signaling pathways activated by OGDR insult.

In this study, we also demonstrated that knockdown of Drp1 by siRNA protects against OGDR induced mitochondrial damage and apoptosis. Drp1 interacts with mutant proteins of neurodegenerative diseases and then fragments mitochondria excessively, ultimately causing neuronal damage [24]. Therefore, Drp1 might also contribute to mitochondrial dysfunction and cell damage in N2a cells through interaction with abnormal protein after OGDR injury. Inhibition of Drp1 has been demonstrated to confer protection in various stress situations. It attenuates mitochondrial fragmentation in cisplatin-induced nephrotoxicity [25], protects the heart against ischemia/reperfusion injury [26], increases retinal ganglion cell survival in acute ischemic mouse retina  [27], provides neuroprotection against glutamate toxicity ischemic brain damage [15, 28], determines the fate of cells after irradiation [29], and protects against chlorpyrifos- (CPF-) induced cytotoxicity [30]. In line with these reports, our data suggest that Drp1 is critical for the morphology and function of mitochondria in N2a cells after OGDR insult. Drp1 inhibition exerts protective effects and is a potential therapeutic target in OGDR injury.

Moreover, our data suggests that overexpression of parkin protects against OGDR induced apoptosis and mitochondrial dysfunction, indicating that parkin is protective in OGDR insult. Parkin protects against misfolded SOD1 toxicity by promoting its aggresome formation and autophagic clearance in amyotrophic lateral sclerosis (ALS) [31]. Parkin has also been shown to exert cytoprotective action in PD and AD by increasing the removal of cellular Abeta through a proteasome-dependent pathway or protecting against the toxicity associated with mutant alpha-synuclein [32, 33]. Therefore, parkin might also confer neuroprotection in OGDR insult by promoting degradation and clearance of toxic proteins through the UPS pathway. Drp1 is a substrate of parkin. We have demonstrated that parkin promotes Drp1 degradation after OGDR insult. Parkin interacts with and subsequently ubiquitinates Drp1, thus promoting its proteasome-dependent degradation [8]. We also found that Drp1 depletion protects against OGDR induced mitochondrial dysfunction and apoptosis. Based on these results, we presume that Drp1 is the downstream effector of parkin. We overexpressed parkin and Drp1 in N2a cells. We found that overexpression of parkin decreased Drp1 expression and cytoprotective action of parkin was abolished by increased expression of Drp1. These results suggest that the neuroprotective effect of parkin in OGDR insult is mediated by decreasing Drp1 expression. Therefore, targeting parkin-Drp1 pathway may provide a novel therapeutic strategy for cerebral ischemia-reperfusion.

In conclusion, our study demonstrates that mitochondria are fragmented, and expression of Drp1 and parkin protein is altered after OGDR insult in mouse N2a cells. The regulated protein level of Drp1 after OGDR insult is mediated by parkin through the ubiquitin proteasome system. Knockdown of Drp1 protects against OGDR induced mitochondrial damage and apoptosis. Moreover, parkin protects against OGDR induced apoptosis and mitochondrial dysfunction by promoting degradation of Drp1, indicating its cytoprotective role in cerebral ischemia-reperfusion injury. Our results showed the beneficial effects of knockdown of Drp1 or overexpression of parkin against OGDR insult, which would pave the way for its potential clinical application. Further efforts may lead to the development of novel therapies for disorders whose etiology is based upon cerebral ischemia-reperfusion injury by targeting parkin-Drp1 pathway.
